# RNA-binding protein immunoprecipitation as a tool to investigate plant miRNA processing interference by regulatory proteins of diverse origin

**DOI:** 10.1186/s13007-018-0276-9

**Published:** 2018-01-31

**Authors:** F. E. Marmisolle, M. L. García, C. A. Reyes

**Affiliations:** Instituto de Biotecnología y Biología Molecular, CCT-La Plata, CONICET - UNLP, calles 47 y 115, 1900, La Plata, Buenos Aires Argentina

## Abstract

**Background:**

Due to the nature of viral RNA genomes, RNA viruses depend on many RNA-binding proteins (RBP) of viral and host origin for replication, dissemination and evasion of host RNA degradation pathways. Some viruses interfere with the microRNA (miRNA) pathway to generate better fitness. The development of an adjusted, reliable and sensitive ribonucleoprotein immunoprecipitation (RIP) assay is needed to study the interaction between RBP of different origin (including viral origin) and miRNA precursors. The method could be further applied to transiently expressed heterologous proteins in different plant species.

**Results:**

Here we describe a modified RIP assay applied to nuclear epitope-tagged proteins of heterologous origin and transiently expressed in *Nicotiana benthamiana*. The assay includes a combination of optimized steps as well as the careful selection of control samples and rigorous data analysis. It has proven efficient to detect and quantify miRNA processing intermediates associated with regulatory proteins.

**Conclusions:**

The RIP method described here provides a reliable tool to study the interaction of RBPs, such as transiently expressed regulatory proteins with lowly represented host RNA, as is the case of miRNA precursors. This modified method was efficiently adjusted to recover nuclear proteins and reduce unspecific background. The purification scheme optimized here for GFP-tagged proteins can be applied to a wide array of RBPs. The subsequent application of next-generation sequencing technologies will permit to sequence and characterize all RNA species bound in vivo by a given RBP.

**Electronic supplementary material:**

The online version of this article (10.1186/s13007-018-0276-9) contains supplementary material, which is available to authorized users.

## Background

MicroRNAs (miRNAs) represent a large family of small RNAs that function as regulators of plant and animal gene expression [1, 2]. Plant primary miRNAs transcripts (pri-miRNAs) are synthesized by RNA pol II and have a 5′cap and 3′poly-A tail [3, 4]. Pri-miRNAs form hairpin-like structures and are sequentially processed by RNAse III-like proteins, namely DICER-like 1 (DCL1) in *Arabidopsis thaliana*, to generate miRNA precursors (pre-miRNAs) and, ultimately, the mature miRNA/miRNA* duplex [5, 6]. Plant and animal viruses can interfere with miRNA-mediated regulation in the host at transcriptional or post-transcriptional level [7–14]. Post-transcriptional alterations may include miRNA processing, accumulation and activity. Recent studies revealed that some viral proteins (VP) interfere with pre-miRNA nuclear export and processing by DICER, as is the case of adenovirus [15] and the Ophiovirus *Citrus psorosis virus* (CPsV) [16]. CPsV 24K protein interacts with pre-miR156*a* and pre-miR171*a* in *Nicotiana benthamiana* plants, causing a higher accumulation of unprocessed precursor species and a concomitant downregulation of mature miRNA species. Consequently, target transcript accumulation is upregulated, possibly leading to differential symptom expression [16].

RNA immunoprecipitation (RIP) is a powerful technique used to detect the association of individual proteins with specific RNA molecules in vivo. This assay has been successfully employed to purify ribonucleoprotein (RNP) complexes from plant tissue extracts, mostly from *Arabidopsis* [17–20]. RNA-binding proteins (RBP)–RNA associations can be identified in RNP complexes involved in well-conserved core RNA processes, but also in specialized processes regardless of whether the interaction occurs in a specific subcellular location like the nucleus. RIP relies on a simple bead- or resin-based affinity purification step that takes advantage of a specific interaction between an antibody and its antigen. The RIP strategies currently employed fundamentally differ in capturing the native RBP either from the wild-type or the epitope-tagged RBPs from transgenic plants. However, little is known about RIP assays using transiently expressed RBP of heterologous origin such as VP [21]. In the case of miRNA precursors, it is well known that they interact with nuclear RBPs like DCL1 in order to be processed, and that the structural features of these precursors are specifically recognized by processing proteins [22]. In plants, miRNA biogenesis is fast and intermediates are in many cases hardly detected [23]. Therefore, methodologies aimed at detecting specific interactions between RBP of heterologous origin and lowly represented miRNA-processing intermediates need to be optimized.

One of the first steps to be adjusted in a RIP assay is tissue preparation, including plant growth, chemical fixation and extraction conditions. Many RBPs are partitioned within the cell. In VP affecting miRNA processing, RBPs are supposed to be located in the nucleus, normally included in the specialized D-bodies [24–26]. Thus, efficient extraction of nuclear and membrane-associated proteins through specific buffer composition should be performed. The selection of a high-affinity antibody for purification of the targeted RNP is another relevant condition to take into consideration. RIP has been successfully implemented in plants using epitope-tagged proteins expressed in transgenic plants and the corresponding commercially available monoclonal antibodies [17, 19, 27]. In transiently expressed heterologous epitope-tagged proteins, RBP level of expression should be high and must be checked. RIP experiments greatly depend on the differentiation between in vivo specific interactions and irrelevant interactions. For instance, an important source of background arises from unspecific interactions of RNPs with the antibody or affinity matrix and therefore RNA binding to co-precipitated proteins leading to false positives. Such background can be reduced by previous clarification of the lysate with a mock preparation or pre-incubating the matrix with an unspecific protein (such as BSA), and also by using stringent washing conditions. Therefore, suitable negative controls are essential. Negative controls for tagging approaches must include tissue which does not express the tagged-RNP under study and/or a tagged-unspecific protein fused to the amino acid sequence required for the interaction with the matrix. Finally, the last step of adjustment in a RIP assay is monitoring the associated candidate target RNAs with the immunoprecipitated RBP. The most sensitive approach is the use of real-time PCR (RT-qPCR) with specific primers. Careful controls are also required in this step since abundant RNAs will inevitably contaminate the affinity preparation. Quantitative data analysis should also be correctly performed to assess *fold enrichment* of the target RNAs between immunoprecipitated (IP) and *Input* fractions.

Here we describe a sensitive RIP method modified from Köster and Staiger [19] and applied to nuclear epitope-tagged proteins of heterologous origin transiently expressed in *N. benthamiana*. Moderate levels of transiently expressed VP have shown to accumulate in *N. benthamiana* leaves, as compared with the high accumulation normally detected in natural infection. The present method provides a feasible and promising tool to be applied in experiments with miRNA processing intermediates.

## Results and discussion

### Modified RIP assay

**Fig. 1:**
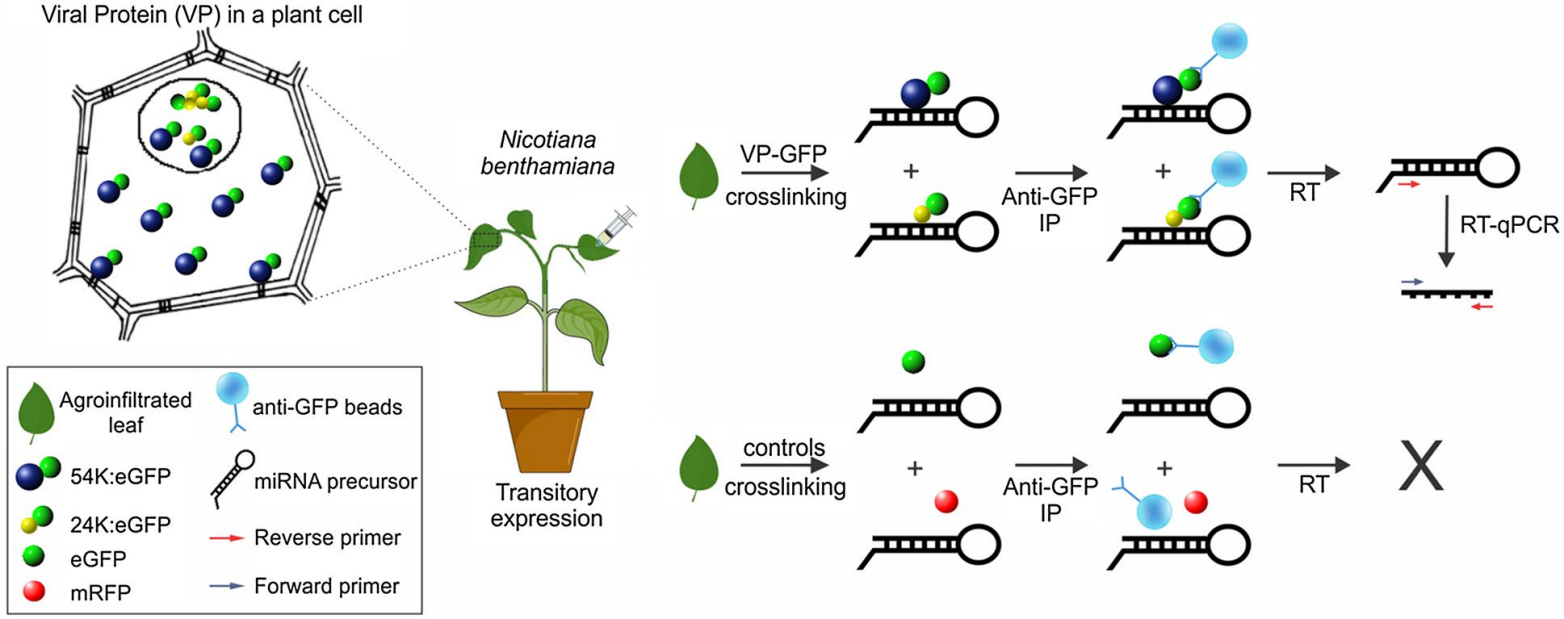
Scheme of RIP experimental approach. VP-GFP (24K or 54K) were transitory expressed in *N. benthamiana* plants. Cross-linked samples were immunoprecipitated using anti-GFP beads and miRNA precursors were analyzed by RT-qPCR

CPsV 24K and 54K proteins are the viral suppressors of RNA silencing (VSR) and have been shown to present affinity for long synthetic double-stranded RNA molecules [28]. These findings, together with the nuclear localization of both proteins, allow us to propose a role for these proteins in the regulation of pre-miRNA processing and miRNA activity [16]. GFP-fused versions of these two viral proteins (VP) were transiently expressed in *N. benthamiana* plants and the RIP method was adjusted to study the differential accumulation of unprocessed miRNA precursors in tissue expressing VP. Non-fused GFP and RFP were included as controls (Fig. 1).

**Fig. 2:**
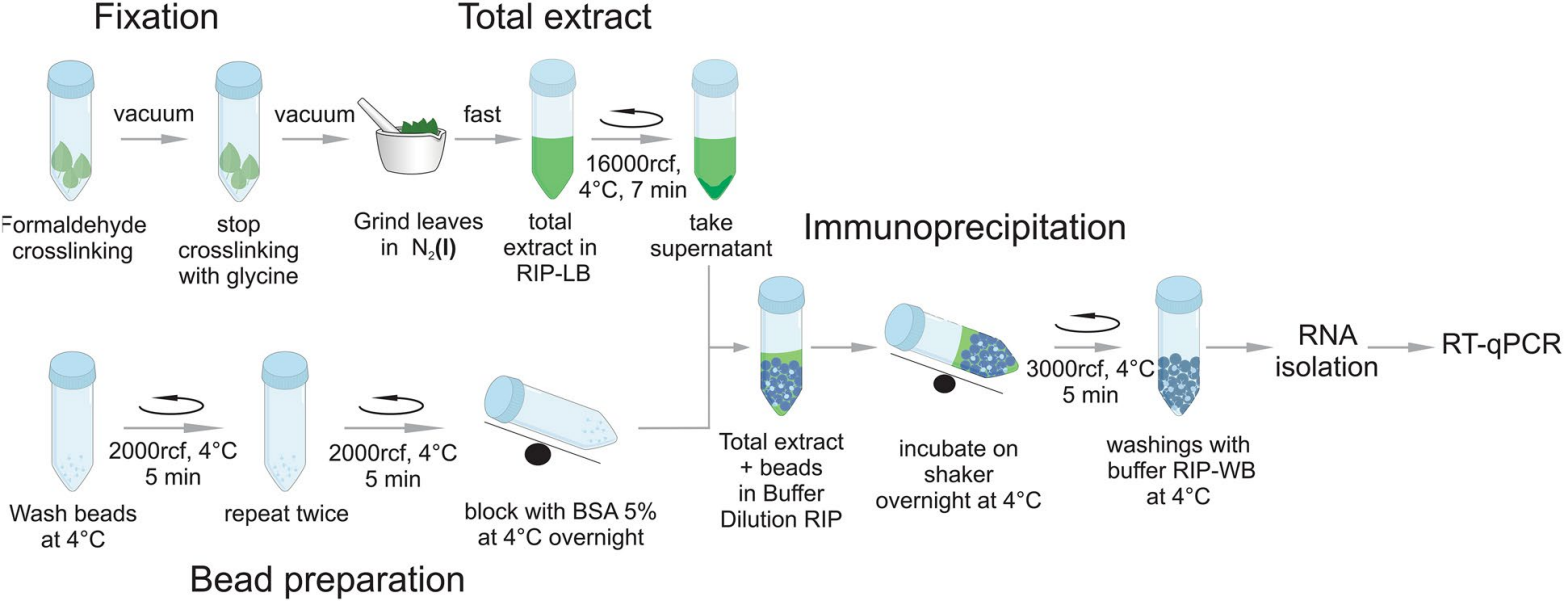
RIP experimental procedure. This outline represents the RIP-qPCR method as described in the text

The RIP method presented here includes a combination of optimized steps (Fig. 2), such as adjusted cross-linking conditions, adequate extraction buffer composition for membrane-bond and partially insoluble proteins, use of a high-quality antibody together with modified immunoprecipitation and elution steps, and inclusion of a highly sensitive RT-qPCR for miRNA precursor detection. A careful selection of negative controls also contributed to the success of the method (Fig. 1).

#### Cross-linking

The first step to increase the sensitivity and reliability of RIP results was the optimization of cross-linking conditions. In vivo cross-linking stabilizes transient and weak RNA–protein complexes, thus allowing the application of more stringent washing conditions. These washing steps reduce contaminants and eliminate unspecific RBP–RNA interactions that may form after cell lysis [29, 30]. In *Arabidopsis*, chemical cross-linking through formaldehyde fixation has led to an 800-fold enrichment of small spliceosomal U2 snRNA precipitated by spliceosomal U2B protein compared with unfixed seedlings [17]. We first checked the expression of VP-GFP (54K and 24K) and control proteins (GFP and RFP) by fluorescence visualization under the microscope (Additional file 1: Fig. S1), and then we performed formaldehyde cross-linking of *N. benthamiana* leaves. To ensure that the cross-linking agent penetrated the plant tissue efficiently and displaced gas at the intercellular space, vacuum was applied and released for short periods. Pressure and time intervals were also adjusted.

#### Extract preparation

The use of rapidly frozen tissue with liquid nitrogen for the preparation of whole cell plant extracts was advantageous for the recovery of intact, uncontaminated RNPs [31]. For instance, RNPs extracted from pulverized frozen leaf tissue were used for the immunoprecipitation of maize chloroplast polysomes translating specific proteins [32] and for the affinity purification of cytosolic mRNAs in *Arabidopsis* associated with ribosomes [27]. We then quickly harvested and froze *N. benthamiana* leaves transiently expressing VP (54K or 24K) and controls in liquid nitrogen.

The interpretation of RIP data can be helped by an understanding of the spatial distribution of the target RBP. For the case of the CPsV 54K and 24K proteins studied here, nuclear localization has already been described [16, 33]. The chosen buffer needed to stabilize different RNPs, particularly nuclear-bond and membrane-included proteins for immunoprecipitation, is likely to differ. Since 24K protein is difficult to solubilize, special care must be taken in buffer composition to effectively recover it. This step was optimized by the addition of mild detergents to the lysis buffer.

#### Antibody description and immunoprecipitation conditions

RBP capturing can be performed by a specific antibody directed against its native form or through the expression of an epitope-tagged version of the RBP. The latter allows the recovery of RBP using highly specific and high-affinity commercially available antibodies for the tag. We used the GFP-TRAP^®^ system (Chromotek, Germany) that consists of agarose beads containing a covalently linked GFP-binding protein, which is the GFP-recognizing domain of a heavy-chain antibody raised in Camelids [34, 35]. The system is extremely stable (up to 70 °C, functional in 2.0 M NaCl or 0.5% SDS) and has a high binding affinity (dissociation constant in the sub-nanomolar range). Additionally, this experimental approach allows the application of the GFP purification scheme optimized here to a wide array of RBPs.

Interactions of RNPs with the antibody or affinity matrix lead to serious background problems. Besides, differentiation of direct from indirect RNA–protein interactions is hindered by RNA binding to co-precipitated proteins. Unspecific binding can be reduced by pre-blocking the beads with a non-cognate protein. For this reason, we included a pre-incubation step of TRAP^®^ beads with BSA. Assays performed without pre-incubation with BSA, showed unspecific interaction of GFP to different RNAs (data not shown). Another consideration is that detergents in the lysis buffer should not exceed 0.2% to avoid unspecific binding to the matrix. Therefore, we diluted the extracted samples in order to keep a low detergent concentration. Washing conditions after immunoprecipitation should also be established carefully to reduce unspecific binding and to avoid the dissociation of specific RNA–protein interactions and RBP–antibody interaction. We then adjusted stringency by including urea (0.5–3 M) in the washing buffer.

#### Cross-linking reversal and RNA isolation

One of the main advantages of formaldehyde cross-linking is its reversibility, since it allows further characterization of immunoprecipitates. Commonly, samples consisting of the washed beads bound to the RBP-RNA complexes are incubated at 70 °C for 45 min to reverse cross-linking [26, 36]. We optimized this step by lowering the temperature and time of incubation. The new conditions were enough to reverse cross-linking and prevent RNA from prolonged incubation and hydrolysis. RNA was then extracted from these samples using Tri Reagent^®^ according to the manufacturer’s protocol.

#### Controls

The use of suitable negative controls is essential for the RIP assay [18]. In the present approach, an aliquot of the cellular lysate was taken before immunoprecipitation (*Input* fraction). This *Input* sample represented the total RNA employed for RIP and served as positive control for the presence of the transcripts under study. Negative controls for immunoprecipitation experiments typically use a unspecific antibody or start with tissues lacking the bait RBP. For tagging approaches, tissue not expressing the tagged isoform is used [19]. We expressed the non-fused GFP driven by identical regulatory elements as in VP-GFP proteins, and both were immunoprecipitated using GFP-TRAP^®^. A comparative analysis of VP-GFP samples or non-fused GFP was then performed by RT-qPCR and VP-GFP was relativized to non-fused samples (see data analysis below). We included RFP immunoprecipitation as an additional negative control of a protein without binding capacity to anti-GFP beads.

#### Pre-miRNA detection and quantitation

Pre-miRNAs are biogenesis intermediates very relevant for studying variations in miRNA processing, such as those reported in CPsV-infected citrus plants [16]. Northern blot is a gold-standard approach that can detect all sizes, ranging from the long pri-miRNA to the mature form, but limited by its low throughput and low sensitivity. qPCR-based approaches are straightforward for measuring the primary transcript, and they can be adjusted to detect low levels of highly structured miRNA precursors [37, 38]. We set conditions to quantify two conserved miRNA precursors (pre-miR156*a* and premiR171*a*) from *N. benthamiana* samples by RT-qPCR and included the well-characterized ubiquitin transcript as internal control [39]. qPCRs were carried out using SYBR-GREEN Master mix (Bio-Rad) and the production of a single PCR product was verified for each primer pair (pre-miR156*a*, pre-miR171*a* and ubiquitin) by detecting a single peak in the melting curve.

#### Data analysis

**Fig. 3:**
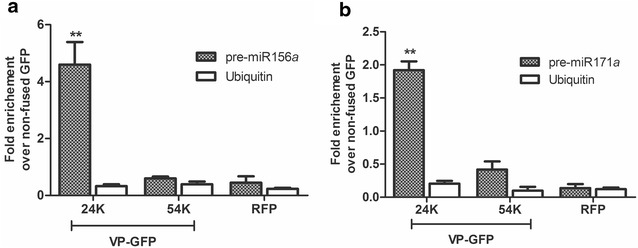
RIP analysis of precursors associated with 24K or 54K GFP-fused proteins from *Citrus psorosis virus* (CPsV) in *Nicotiana benthamiana*. RT-qPCR was performed to determine the accumulation levels of *N. benthamiana* pre-miR156*a* (**a**) or pre-miR171*a* (**b**). *Fold enrichment* of the immunoprecipitated (RIP) precursors was calculated as 2^(−ΔΔCt [RIP/background])^. Mean values and standard errors of three independent experiments are shown. Statistical analysis was performed using a two-tailed paired *t* test; * and ** indicate significant differences from RFP control sample at P < 0.05 and P < 0.01 values, respectively. *GFP* green fluorescent protein, *RFP* red fluorescent protein

We calculated *fold enrichment* of each RIP reaction from qPCR data (see formulas below). We processed four samples: VP 24K and 54K and two controls, non-fused GFP and RFP. We first normalized the Ct value of the four IP RNA to the *Input* RNA fractions to eliminate possible differences in RNA sample preparation (ΔCt normalized RIP). Presenting RIP signals as *fold enrichment* of the studied proteins over signals in the non-fused GFP control (unspecific background) would be the most convenient way, accounting also for the specificity of the procedure. Thus, to calculate *fold enrichment,* the normalized RIP fraction value (ΔCt normalized RIP of 24K, 54K or RFP) was first normalized to unspecific background (ΔCt normalized of non-fused GFP sample), obtaining the ΔΔCt. Finally, the linear conversion of this ΔΔCt rendered *fold enrichment,* which was plotted for 24K, 54K and RFP (Fig. 3). *Fold enrichment* of the qPCR internal control, ubiquitin, was also calculated in all samples, and no significant enrichment was observed (Fig. 3).

$$\begin{aligned} &\Delta {\text{Ct}}\,\left[ {{\text{Normalized}}\,{\text{RIP}}} \right] = ({\text{Ct}}\,\left[ {\text{IP}} \right]{-}\left( {{\text{Ct}}\,\left[ {\text{Input}} \right]{-}{\text{Log}}2\,\left( {{\text{Input/IP}}\,{\text{dilution}}\,{\text{factor}}} \right)} \right) \\ & {\Delta \Delta }{\text{Ct}}\,\left[ {\text{RIP/GFP}} \right] =\Delta {\text{Ct}}\,\left[ {{\text{Normalized}}\,{\text{RIP}}} \right]{-}\Delta {\text{Ct}}\,\left[ {{\text{Normalized}}\,{\text{non-fused}}\,{\text{GFP}}} \right] \\ & {\text{Fold}}\,{\text{enrichment}} = 2^{{( - {\Delta \Delta }{\text{Ct}}\,[{\text{RIP/GFP}}])}} \\ \end{aligned}$$

#### Protein analysis

Immunoprecipitated proteins were recovered from TriReagent^®^ organic phase. The expression, size and integrity of the viral and control proteins were confirmed by Western blot in the *Input* and IP fractions (Additional file 2: Fig. S2). Silver-stained polyacrylamide gels were also run to check immunoprecipitation progress (Additional file 3: Fig. S3).

## Methods

### Materials and reagents

#### Plant growth and infiltration

*Nicotiana benthamiana* plants were cultivated in pots, under long days conditions (16 h light and 8 h darkness) at 24 °C in growing chambers. They were used to agroinfiltrate when they reached the 4 leaves. *Agrobacterium tumefaciens* strain GV3101 carrying VP-GFP, non-fused GFP or RFP constructs were used at OD_600_ between 0.2 and 0.4. Infiltration was performed by pressing a syringe (no needle) containing the cultures on the underside of the leaf. Samples were collected 4 days after infiltration.

#### Reagents and solutions

Bovine Serum Albumin—BSA (SIGMA-ALDRICH, Cat. No. B2518).

Calcium chloride—CaCl_2_ (SIGMA-ALDRICH, Cat. No. C1016).

Dithiothreitol—DTT (SIGMA-ALDRICH, Cat. No. D-9779).

Ethylenediamine tetraacetic acid—EDTA (SERVA, Cat. No. 11278).

Formaldehyde solution (SIGMA-ALDRICH, Cat. No. F8775).

GFP-Trap^®^_A (Chromotek, Alemania. http://www.chromotek.com/products/nano-traps/gfp-trap/gfp-trapr-a/).

Glycine (SIGMA-ALDRICH, Cat. No. G8898).

Magnesium chloride·6H_2_O—MgCL_2_·6H_2_O (SERVA, Cat. No. 28305.01).

M-MLV Reverse Transcriptase—MMLV-RT (Promega, Cat. No. M1701).

M-MLV Reverse Transcriptase 5X Reaction Buffer—MMLV buffer (50 mM Tris–HCl (pH 8.3 at 25 °C), 75 mM KCl, 3 mM MgCl2 and 10 mM DTT—Promega, Cat. No. M531A).

Phenylmethanesulfonyl fluoride—PMSF (SIGMA-ALDRICH, Cat. No. P7626-1G).

RNase inhibitor—RNAsin (Genbiotech, SRL, Cat. No. PE3013).

RQ1 RNase-Free DNase (Promega, Cat. No. M6101).

RQ1 DNase 10X Reaction Buffer—DNase Buffer (400 mM Tris–HCl (pH 8.0), 100 mM MgSO_4_ and 10 mM CaCl_2_—Promega, Cat. No. M198A).

Sodium chloride—NaCl (SERVA, Cat. No. 15585).

Sodium Deoxycholate (SIGMA-ALDRICH, Cat. No. D6750).

Sodium dodecyl sulfate—SDS (SERVA, Cat. No. 151-21-3).

Stop Solution: 20 mM EGTA (pH 8.0) (Promega, Cat. No. M199A).

SYBR-GREEN Master mix (Bio-Rad, Cat. No. 1708882).

TRI-Reagent^®^ (Molecular Research Center, Inc.).

Tris [hydroxymethyl]aminomethane (SIGMA-ALDRICH, Cat. No. T-1378).

Triton X-100 (SERVA, Cat. No. 37240).

Urea (SERVA, Cat. No. 24524).

*RIP Lysis Buffer* (RIP-LB): 20 mM Tris–HCl (pH = 7.5), 150 mM NaCl, 1 mM MgCl_2_, 1 mM CaCl_2_, 0.1% SDS, 1% Sodium Deoxycholate, 1% Triton X-100. Before using add: 5 mM PMSF and 5 mM DTT.

*RIP Washing Buffer* (RIP-WB): 50 mM Tris–HCl (pH = 7.5), 500 mM NaCl, 4 mM MgCl_2_, 0.5% Sodium Deoxycholate, 0.1% SDS, 2 M Urea. Before using add 2 mM DTT.

*RIP dilution Buffer* (RIP-DB): 10 mM Tris–HCl (pH = 7.5), 150 mM NaCl, 0.5 mM EDTA.

**RIP Procedure**

***Day 1***

**Preparation of GFP-TRAP**

[Note: keep tubes on ice during all processing]

Washes:

1. Wash 30 μl of GFP-TRAP with 1 ml of cold RIP-LB for 5 min at 4 °C.
2. Centrifuge 5 min at 2000 rcf at 4 °C. Remove supernatant.
3. Repeat washing twice.

[Note: be aware that beads precipitate (white pellet) when spinning, if not repeat the spin].

Blocking:

1. Add 500 μl of RIP-LB + BSA 5% to the beads.
2. Incubate overnight in shaker at 4 °C.
3. At the time of use, centrifuge for 3 min at 2000 rcf at 4 °C. Remove supernatant.

***Day 2***

**Fixation**

1. Place five agroinfiltrated *N. benthamiana* leaves* in a 15 ml tube.
2. Add 10 ml of 1% formaldehyde (liquid level should cover the leaves).
3. Apply vacuum with pump, 60–64 cmHg for 15 min (5 times of 3 min).
  [Note: check that all leaves have absorbed the liquid].
4. Discard formaldehyde and replace it with 10 ml of 125 mM glycine.
5. Apply vacuum with pump, 60–64 cmHg for 15 min (5 times of 3 min).
6. Remove the solution.
7. Wash the leaves 4 times with RNase-free water at 4 °C.
8. Remove the liquid. Blot the leaves carefully on absorbent paper.

*VP-GFP, non-fused GFP or RFP expression was previously checked by fluorescent microscopy using a *Nikon eclipse* e200 microscope with a 40× objective and EN/GFP 41017 and G2-A filters for GFP and RFP respectively. Images were processed with ImageJ software.

**Preparation of total extract**

1. Add 500 μl of RIP-LB in a 1.5 ml tube and place on ice. Add 50 U/tube of RNase inhibitor.
2. Grind 5 leaves in liquid nitrogen to fine powder. Add 0.5 g of extract to each tube containing RIP-LB.
3. Vortex 15 min at 4 °C.
4. Incubate for 10 min on ice.
5. Centrifuge 7 min at 16000 rcf at 4 °C.
6. Transfer the supernatant to a new tube. Spin again.

[Note: this step is repeated once again in order to obtain a clearer supernatant].

**Immunoprecipitation**

1. Add to the washed beads (in order): 550 μl of RIP-DB, 50 μl of the total extract supernatant and 40 U of RNase inhibitor.
2. *INPUT* CONTROL: Add to a new 1.5 ml tube: 550 μl of RIP-DB and 50 μl of the total extract supernatant and 40 U of RNase inhibitor.
3. Incubate both groups of tubes overnight at 4 °C with softly shaking.

***Day 3***

1. Centrifuge tubes with beads 5 min at 3000 rcf and 4 °C.
2. Take the supernatant. Freeze rapidly in liquid nitrogen and store at − 80 °C.
3. Add 1 ml of RIP-WB at 4 °C to the beads.
4. Centrifuge 5 min at 3000 rcf and 4 °C. Remove the supernatant carefully and discard.
  [Note: beads are observed as a little defined white precipitate. Repeat centrifugation may be necessary].
5. Repeat washing 4 more times.
6. Finally, wash with 1 ml of cold RIP-LB.
7. Centrifuge 5 min at 3000 rcf and 4 °C. Discard the supernatant.

**Isolation of RNA**

1. Add 400 μl of TRI-Reagent^®^ to the tubes with the beads and to the *INPUT* CONTROL.
2. Vortex 15 s.
3. Incubate 5 min at 55 °C. Mix the tubes with your hand every 30 s.
4. Add 100 μl of chloroform.
5. Continue procedure as described by de manufacturer.
6. Resuspend the *Input* pellets in 20 μl of RNase-free water and the immunoprecipitate pellets in 8 μl of RNase-free water.

[Note: RNA may be in very low concentration in the immunoprecipitate pellets. It is convenient not to measure it. In the case of *Inputs* the RNA concentration was estimated from the absorbance quantification at 260 nm measured in a *ND*-*1000 Spectrophotometer* (NanoDrop Technologies, Inc.)].

**DNase treatment**

1. Take the 8 μl of the immunoprecipitate fraction and 2 μg of the *Inputs*.
2. Add 1 μl DNase Buffer and 1 μl RQ1 RNase-Free DNase. Add RNase-free water to a final volume of 10 μl. Incubate at 37 °C for 30 min.
3. Add 1 μl of Stop Solution. Incubate at 65 °C for 10 min.

**Reverse transcription**

1. Take 4 μl of the sample treated with DNase. Add 0.64 μl of the reverse primer (100 mM) and 9.06 μl of RNase-free water. Incubate at 80 °C for 5 min. Quickly put in ice-water for 5–10 min.
2. Incubate for 5 min at 60 °C.
3. Add 5 μl of MMLV buffer, 5 μl dNTPs, 0.3 μl RNase inhibitor and 1 μl MMLV-RT. The final reaction volume was 25 μl. Incubate at 42 °C for 60 min and then 15 min at 70 °C.

### RT-qPCR analysis

For RT-qPCR analysis of pre-miR156*a* and pre-miR171*a*, we synthesized first-strand cDNA using MMLV from total DNase-treated RNA. Specific primers [pre156aF 5′-TGACAGAAGAGAGTGAGCAC-3′ pre156aR and 5′-GCTGACAGAAAGAGCAGTGA-3′ for pre-miR156*a*, and NB.pre171aF 5′-TATTGGTGCGGTTCAATGAGA-3′ and NB.pre171aR 5′-GGCACGGCTCAATCAAAAAG-3′ for pre-miR171*a*] were used. cDNA was then used as a template for qPCRs. qPCR was performed with an iCycler iQ (Bio-Rad) and SYBR-GREEN Master mix (Bio-Rad) in a reaction held at 95 °C for 10 min, then 44 cycles of 20 s at 95 °C, 30 s at 48 °C and 20 s at 72 °C, followed by melting curve. The presence of a unique product of the expected size was verified on ethidium bromide-stained agarose gels. The absence of contaminant genomic DNA was confirmed in reactions with DNase-treated RNA as the template. *N. benthamiana* ubiquitin amplification was used to normalize the amount of template cDNA. For this, the primers used were NB.UBQF 5′-ATCCACCCGACCAGCAGAG-3′ and NB.UBQR 5′-TAGAAACCACCACGGAGACG-3′. Three independent experiments and three biological replicates per experiment were performed. The reproducibility of the assay was monitored by running technical triplicates. Data analysis was performed as described above.

### Protein analysis

VP-GFP, GFP and RFP in *Input* and IP fractions were detected by Western blot with anti-GFP JL-8 monoclonal antibody (BD Biosciences Clontech, USA) and anti-mRFP 3F5 monoclonal antibody, respectively (Chromotek, Germany). Horseradish Peroxidase conjugated anti-mouse (Bio-Rad, USA) was used as secondary antibody. Silver-stained polyacrylamide gels (10%) were run of *Input* and IP fractions as previously described [40]. Protein sizes were estimated using the ECL™ Rainbow™ Marker Full-range protein ladder 12–225 kDa (Amersham™). Different exposure times were taken according to the protein accumulation.

## Additional files

**Additional file 1: Fig. S1.** Fluorescent microscopy of VP 24K (**a**), 54K (**b**) and controls GFP (**c**) and RFP (**d**). Nuclear and cytoplasmic localization of the proteins at 3 days post-agroinfiltration in *Nicotiana benthamiana* epidermal cells. Left bottom insets show a detail of the nuclear localizations. Scale bar, 10 μm.

**Additional file 2: Fig. S2.** Western blot analysis of transiently expressed 24K or 54K in *Nicotiana benthamiana* plants before (*Input*) and after (IP) RNA immunoprecipitation. The two fractions were analyzed using anti-green fluorescent protein (α-GFP) antibodies for the GFP-fused viral proteins (left panels) or anti-red fluorescent protein (α-RFP) antibodies for the RFP control (right panels). Different exposure times are indicated under IP panels. Coomassie blue-stained sodium dodecylsulfate-polyacrylamide gel electrophoresis (SDS-PAGE) is shown in the lower panel of the *Input* fraction as a loading control.

**Additional file 3: Fig. S3.** Silver-stained SDS-PAGE analysis in *Inputs* and RNA immunoprecipitation (IP). **a** Black arrow indicates GFP. Amounts loaded for *Input* and IP correspond to 10 and 20 mg of the initial tissue respectively. Asterisks indicate artifact signal from the staining. **b** White arrow indicates 24K-GFP fusion (24K) and white dash indicated free GFP. Amount loaded for *Input* and IP corresponds to 10 mg of the initial tissue. *INPUT* (2) corresponds to a shorter time of staining respect to *INPUT* (1).
