# Supplementary figures and images for: RNA-binding protein immunoprecipitation as a tool to investigate plant miRNA processing interference by regulatory proteins of diverse origin
Source: Plant Methods. 2018 Jan 31;14:9. doi: 10.1186/s13007-018-0276-9 (PMC5791195; doi:10.1186/s13007-018-0276-9)

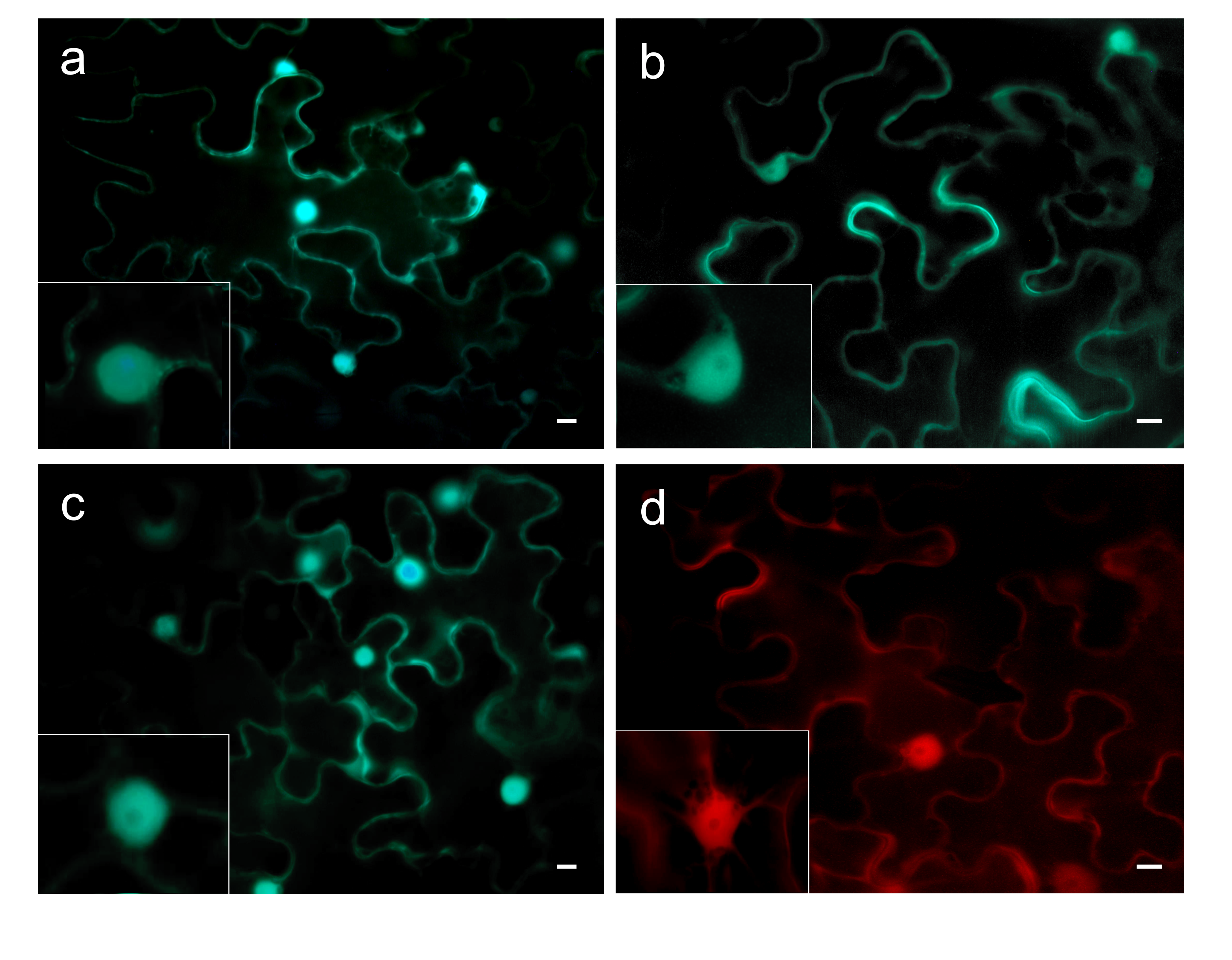

Supplement: Supplementary file 1 — Additional file 1: Fig. S1. Fluorescent microscopy of VP 24K (a), 54K (b) and controls GFP (c) and RFP (d). Nuclear and cytoplasmic localization of the proteins at 3 days post-agroinfiltration in Nicotiana benthamiana epidermal cells. Left bottom insets show a detail of the nuclear localizations. Scale bar, 10 μm. [file 13007_2018_276_MOESM1_ESM.tif]

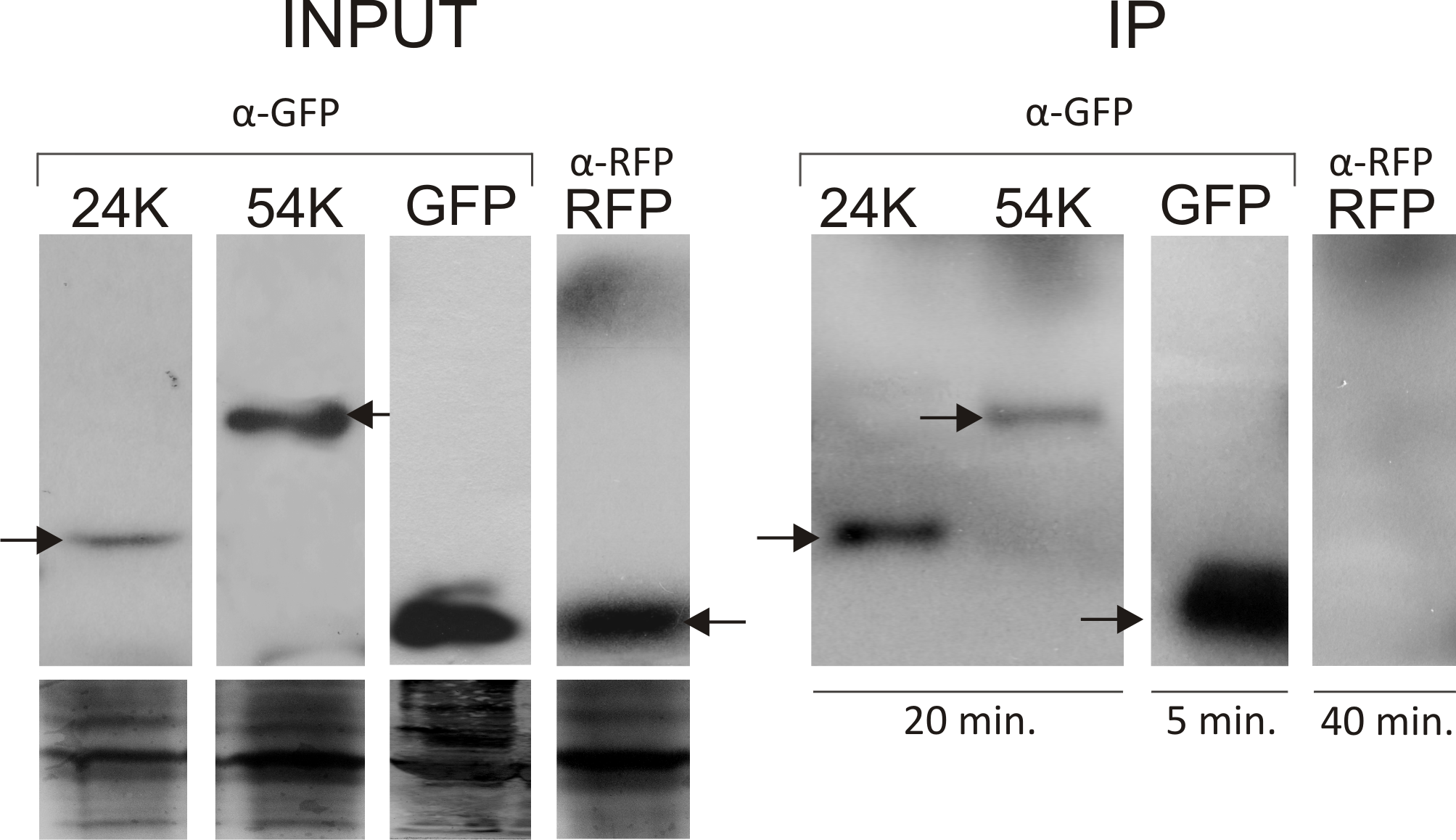

Supplement: Supplementary file 2 — Additional file 2: Fig. S2. Western blot analysis of transiently expressed 24K or 54K in Nicotiana benthamiana plants before (Input) and after (IP) RNA immunoprecipitation. The two fractions were analyzed using anti-green fluorescent protein (α-GFP) antibodies for the GFP-fused viral proteins (left panels) or anti-red fluorescent protein (α-RFP) antibodies for the RFP control (right panels). Different exposure times are indicated under IP panels. Coomassie blue-stained sodium dodecylsulfate-polyacrylamide gel electrophoresis (SDS-PAGE) is shown in the lower panel of the Input fraction as a loading control. [file 13007_2018_276_MOESM2_ESM.tif]

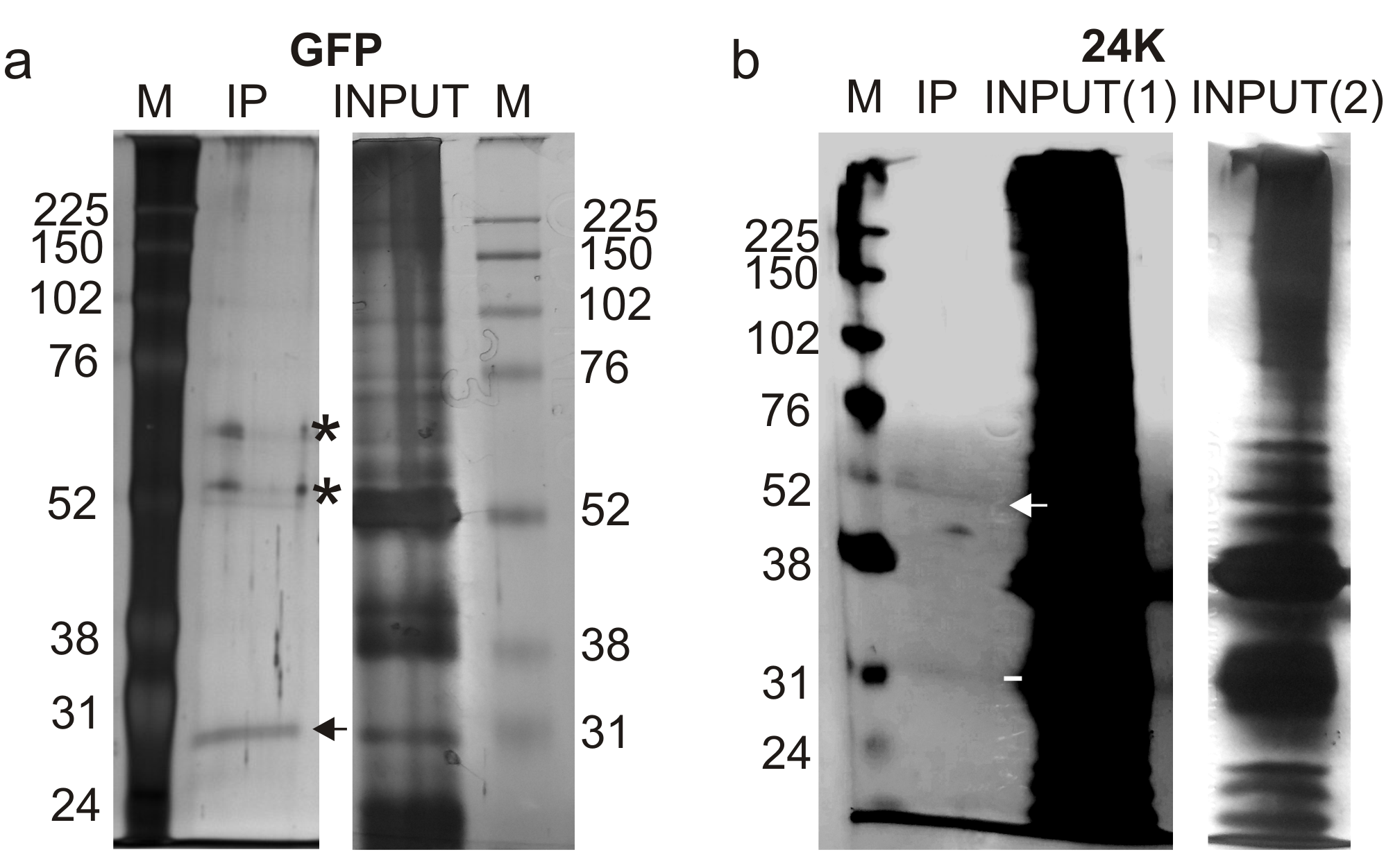

Supplement: Supplementary file 3 — Additional file 3: Fig. S3. Silver-stained SDS-PAGE analysis in Inputs and RNA immunoprecipitation (IP). a Black arrow indicates GFP. Amounts loaded for Input and IP correspond to 10 and 20 mg of the initial tissue respectively. Asterisks indicate artifact signal from the staining. b White arrow indicates 24K-GFP fusion (24K) and white dash indicated free GFP. Amount loaded for Input and IP corresponds to 10 mg of the initial tissue. INPUT (2) corresponds to a shorter time of staining respect to INPUT (1). [file 13007_2018_276_MOESM3_ESM.tif]
